# Recurrent Vocal Fold Paralysis and Parsonage-Turner Syndrome

**DOI:** 10.1155/2013/763201

**Published:** 2013-10-31

**Authors:** Marcus Vinicius Pinto, Lucia Joffily, Maurice Borges Vincent

**Affiliations:** ^1^Department of Neurology, Clementino Fraga Filho University Hospital, Federal University of Rio de Janeiro, 21941-590 Rio de Janeiro, Brazil; ^2^Department of Otorhinolaryngology, Clementino Fraga Filho University Hospital, Federal University of Rio de Janeiro, 21941-590 Rio de Janeiro, Brazil

## Abstract

*Background*. Parsonage-Turner syndrome, or neuralgic amyotrophy (NA), is an acute brachial plexus neuritis that typically presents with unilateral shoulder pain and amyotrophy but also can affect other peripheral nerves, including the recurrent laryngeal nerve. Idiopathic vocal fold paralysis (VFP) represents approximately 12% of the VFP cases and recurrence is extremely rare. *Methods and Results*. We report a man with isolated recurrent unilateral right VFP and a diagnosis of NA years before. *Conclusions*. We emphasize that shoulder pain and amyotrophy should be inquired in any patient suffering from inexplicable dysphonia, and Parsonage-Turner syndrome should be considered in the differential diagnosis of idiopathic VFP.

## 1. Introduction

Neuralgic amyotrophy (NA), or Parsonage-Turner syndrome, is an acute brachial plexus neuritis that typically presents with unilateral severe shoulder pain followed by patchy paresis and atrophy [1–3]. Although the pathophysiology of NA remains obscure, a predisposition for an acute immune attack towards the peripheral nerves following immunization, infections, exercise, labor, trauma, or surgery may occur [3, 4]. The incidence of 2-3 cases per 100,000 [5, 6] a year may not reflect the real figures as NA is for a certainty underdiagnosed. Idiopathic neuralgic amyotrophy (INA) is ten times more common than hereditary neuralgic amyotrophy (HNA) [4]. The prognosis is generally good, as 90% of the patients are almost fully recovered after 3 years [2].

The NA phenotype may vary to a large extent. Other segments of the peripheral nervous system (PNS) may be affected, such as the lumbosacral plexus, phrenic nerve, and the recurrent laryngeal nerve. The upper trunk of the brachial plexus is the most affected section, but involvement of single nerves, as the suprascapular, axillary, and anterior interosseus nerves, is common. The involvement of the recurrent laryngeal nerve is rare, particularly in INA [4, 7, 8]. There are only two reports of NA attacks presenting as vocal fold paralysis without pain or weakness [9, 10].

Unilateral vocal fold paralysis (VFP) is a disorder caused by dysfunction of the brainstem nuclei, the vague nerve, or the recurrent laryngeal nerve supplying the involved side of larynx. Nonlaryngeal malignancy and iatrogenic nerve injury are the major causes of unilateral VFP, while idiopathic cases correspond to approximately 12% of the patients [11]. Recent reports have shown a decrease in idiopathic cases, probably reflecting better diagnostic capabilities [12]. The symptoms of unilateral VFP are related to immobility of the vocal fold causing glottic insufficiency. The primary symptom of unilateral VFP is dysphonia, expressed as hoarseness. With time, it can progress to aspiration of liquids and, in some cases, exertional dyspnoea [13]. Many cases of idiopathic unilateral VFP recover spontaneously leading to improvement in voice quality [11–13]. VFP recurrence is extremely unusual. There has been only one case of HNA with recurrent isolated VFP described in a child so far [9].

We report a patient with classical INA years before the development of recurrent unilateral VFP. To the best of our knowledge, this is the first reported case of NA with isolated recurrent unilateral VFP.

## 2. Case Report

A 24-year-old right-handed man was referred to the Neurology by the Ear, Throat and Nose Department because of recurrent idiopathic right VFP with a diagnosis of INA made nine years before. When he was 15, he presented with acute pain in the right shoulder with right upper arm weakness two weeks after an upper respiratory tract infection. The neurological examination one month later showed winged scapula, amyotrophy of the trapezius and deltoid muscles, and hypoesthesia over the right deltoid region. The electromyography (EMG) depicted positive sharp waves and fibrillation potentials with reduced recruitment in right trapezius and deltoid muscles with no abnormalities in the left arm muscles. The neuroconduction studies were normal. In the absence of NA family history, the diagnosis of INA was suggested after cervical spine magnetic resonance imaging (MRI) and blood tests proved normal. He was clarified about the diagnosis and started physical therapy. After 6 months, he was completely recovered. 

Four years later, he presented with acute dysphonia in the absence of dyspnoea, dysphagia, shoulder pain, weakness, or any other symptom 10 days after having an upper airway infection. Fibre optic laryngoscopy showed immobility of the right vocal fold in paramedian position, suggesting right recurrent laryngeal nerve paralysis. Chest and cervical computerized tomography (CT), brain MRI, and blood tests were unremarkable, and so he received the diagnosis of idiopathic right VFP. He was treated with prednisone 1 mg/kg for one month without improvement. Four months later, his voice was normal again and the video laryngoscopy showed normal vocal fold function. 

**Figure 1:**
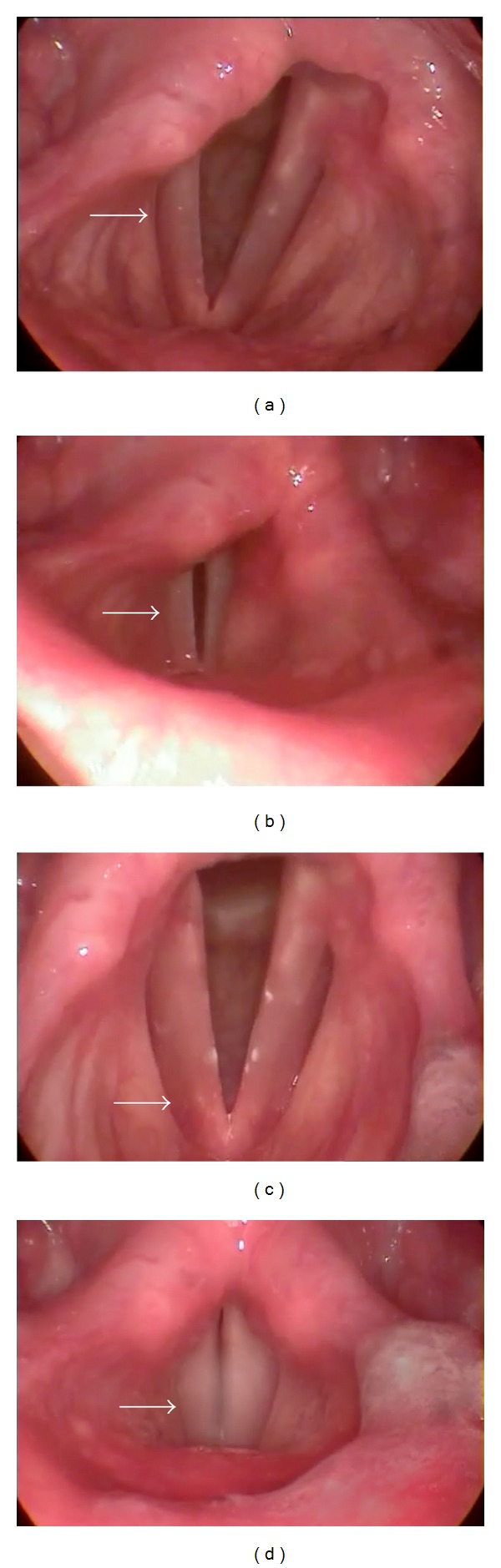
Video laryngoscopy showing immobility of the right vocal fold in a paramedian position, either in abduction (a) or in adduction (b) of the glottis. After 8 months, showing normal mobility of both vocal fold, either in abduction (c) or adduction (d) of the glottis.

Five years after the first VFP, he started a new episode of acute dysphonia while he was singing at a band rehearsal. The video laryngoscopy showed again immobility of the right vocal fold in paramedian position (Figure 1). He had started to sing in this band the month before, and two weeks prior to this event he had an acute tonsillitis. The neurological examination was normal except for dysphonia. After normal chest and cervical CT scans, cervical spine and brain MRI exams, and negative blood tests for virus infections, vasculitis, and collagen diseases, the diagnosis of idiopathic right VFP was once again made. As in the first episode, he was treated with prednisone for one month without any improvement. After 8 months, he was fully recovered, and the video laryngoscopy showed normal vocal fold function (Figure 1).

## 3. Discussion

Parsonage and Aldren Turner described in 1948 a typical clinical syndrome of severe unilateral shoulder pain followed by amyotrophy of the shoulder girdle muscles, often preceded by infection, trauma, immunization, surgery, or trauma [1]. In 1972, Tsiaris et al. called attention to the brachial plexus involvement, predominantly the upper plexus, based on clinical and EMG findings in 99 patients [2]. Evidence emerged showing that not only the brachial plexus is affected but also individualized nerves and other PNS structures outside the arms may be involved. EMG is important for the identification of affected PNS structures and in addition to cervical spine and brachial plexus MRI help for the differential diagnosis of NA [3, 4, 7, 8]. The pathophysiology remains unknown, despite of sturdy progresses in nerve pathology, genetic, and epidemiological studies during the last decades.

Brachial plexus biopsies performed in four INA patients showed that inflammatory mononuclear cells infiltrate surrounding endoneurial and epineurial vessels [14]. Klein et al. demonstrated inflammatory infiltrates in 2 out of 4 radial superficial nerve biopsies in HNA patients [15]. These findings, associated with the fact that more than 50% of patients have an immune event before the NA attack, strongly suggest an immune mediated mechanism in the pathophysiology of NA [4].

In 2005, three mutations in the gene SEPT9 located to the chromosome 17q25 were described in HNA kindred's [16]. This gene was found to be abnormal in 55% of the HNA families [4], indicating that other loci are still to be discovered. The human septin family is composed of 14 loci, SEPT1-SEPT14, which encode septin. These are GTP-binding proteins that form hetero-oligomeric complexes that are pivotal in cell cytoskeleton and division [17]. This discovery supports the hypothesis that NA patients are susceptible to peripheral nerve injury as 10% of the patients have an antecedent history of unusual physical exercise [3]. Some authors also hypothesize that the preference for the brachial plexus is based on its high mobility that weakens the blood-nerve barrier, making it more susceptible to immune factors and cells contact within the nerves [4]. NA has in all probability a complex pathophysiology that involves genetic predisposition, biomechanical factors, and autoimmune mechanisms.

The largest NA series was published in 2006 (199 INA and 47 HNA patients), shedding new light on this intriguing disorder [3]. The clinical picture might be bilateral in 29% of the subjects, and recurrence may occur in, respectively, 75% and 26% of HNA and INA cases, within 5–10 years. In the HNA group, nerves outside the brachial plexus are affected in 56% of the patients, compared with 17% in INA. The lumbosacral plexus is the most common structure involved (33% in HNA, 17% in INA) outside the brachial plexus, followed by the recurrent laryngeal nerve (19% in HNA, 2% in INA) and phrenic nerve (14% in HNA, 7% in INA) [3]. These patients with atypical presentations are included in the NA extended spectrum group [4, 7, 8].

There are a few reports of isolated phrenic nerve paralysis as a unique manifestation of Parsonage-Turner syndrome [18, 19]. Tsao et al. reviewed medical records of 33 cases of idiopathic phrenic nerve paralysis and found that 17 patients had clinical features of NA [18]. A patient that developed unilateral recurrent laryngeal nerve palsy one week before shoulder pain and amyotrophy was recently reported [20]. Wurmser and Kaeser described a patient who developed unilateral recurrent laryngeal nerve palsy 7 months before shoulder pain and amyotrophy [10]. Lechinsky-Silver et al. reported a case of a child with HNA with recurrent isolated bilateral VFP between typical neuralgic amyotrophy attacks [9]. We described a case of isolated recurrent VFP in an INA patient.

The present patient had a classical NA attack when he was 15 years old, affecting the right axillary and spinal accessory nerves. Besides, he had two episodes of isolated right recurrent laryngeal nerve palsy, four and nine years after his first NA attack. All the episodes were preceded by a viral infection. The second vocal fold paralysis occurred when he was singing, a strenuous exercise for his vocal cords. Probably, viral infections and vocal fold exercise (latter episode) functioned as triggers for an immune attack against the peripheral nerves in this predisposed individual. As typically for NA attacks, the second VFP episode required twice as much time to improve. Short courses of prednisone were ineffective in both VFP episodes. Although there is no evidence from randomized trials on any type of treatment, steroids given during the first month of an attack probably shorten pain and accelerate recovery in some patients. Until now, no genetic mutation has been described in these recurrent INA patients [4].

Notwithstanding the lack of a biological marker reassuring a link between recurrent isolated unilateral VFP and NA, there is sturdy clinical evidence indicating that the recurrent VFP is a consequence of NA in this case.

No other recurrent unilateral VFP were reported in the adult as an isolated manifestation of NA. We emphasize that shoulder pain and amyotrophy should be inquired in any patient suffering from inexplicable dysphonia, and Parsonage-Turner syndrome should be considered in the differential diagnosis of idiopathic VFP. Ear, nose, and throat physicians might consider a neurology consultation in such cases. 

## Abbreviations

NA: Neuralgic amyotrophy
INA: Idiopathic neuralgic amyotrophy
HNA: Hereditary neuralgic amyotrophy
VFP: Vocal fold paralysis
PNS: Peripheral nervous system
EMG: Electromyography
MRI: Magnetic resonance imaging
CT: Computerized tomography.
